# EFFECTS OF INTRAPERITONEAL GLUTAMINE IN THE TREATMENT OF EXPERIMENTAL SEPSIS

**DOI:** 10.1590/0102-672020190001e1431

**Published:** 2019-04-29

**Authors:** Emanuele Therezinha Schueda STONOGA, Roberta Zawadzki BUENO, Thais Ayumi NAGANO, Vanessa MARTINS, Sérgio Luiz ROCHA

**Affiliations:** 1 Department of Operative Technique and Experimental Surgery of the Pontifical Catholic University of Paraná, Curitiba, PR, Brazil.

**Keywords:** Sepsis, Glutamine, Peritonitis, Rats, Intestines, Sepse, Glutamina, Peritonite, Ratos, Intestinos

## Abstract

**Background:**

Sepsis is an important public health issue and is associated with high treatment costs and high mortality rates. Glutamine supplementation has proven to be beneficial to the functions of the immune system, acting beneficially in the evolution of patients in severe catabolic states.

**Aim:**

To evaluate the effect of glutamine supplementation via intraperitoneal in rats, induced sepsis, considering the following organs: intestines, liver, kidneys and lungs.

**Methods:**

Male Wistar rats subjected to sepsis by ligature and cecal puncture were divided into two groups: control C (n=6) and glutamine G (n=11), in which were administered dipeptiven 20% at a dose of 2 ml/kg/day (equivalent to 0.4g N(2)-L-alanyl-L-glutamine/kg) intraperitoneally 48 h prior to sepsis induction. After 48 h they were euthanized and intestine, liver, lung and kidney were removed for histological analysis.

**Results:**

Intestinal epithelial desquamation of the control group was more intense compared to the glutamine group (p=0.008). In the kidneys, degenerative tubular epithelial changes were less severe in the animals that received glutamine (p=0.029). Regarding to the liver, glutamine group showed lower levels of cell swelling than the control group (p=0.034). In the lung there were no results with statistical significance.

**Conclusion:**

Prior intraperitoneal supplementation with glutamine in experimental animals is able to reduce the damage to the intestinal mucosa, to the kidneys and liver’s histoarchitecture.

## RESUMO

**Racional:**

A sepse é importante problema de saúde pública, sendo relacionada com altos custos de tratamento e elevadas taxas de mortalidade. A suplementação de glutamina tem provado ser benéfica às funções do sistema imune, atuando em estados catabólicos graves.

**Objetivo:**

Avaliar o efeito da suplementação de glutamina via intraperitoneal em ratos induzidos à sepse.

**Método:**

Foram utilizados ratos Wistar submetidos à sepse por ligadura e punção do ceco, separados em grupo controle C (n=6) e glutamina G (n=11), aos quais foram administrados dipeptiven a 20% com dose de 2 ml/kg/dia (equivalente a 0,4 g N(2)-L-alanil-L-glutamina/kg), via intraperitoneal, 48 h antes da indução da sepse. Após 48 h todos os animais foram submetidos à eutanásia e intestino, fígado, pulmão e rim foram retirados para análise histológica.

**Resultados:**

No intestino a descamação epitelial do grupo controle foi mais intensa em comparação ao da glutamina (p=0,008). Nos rins, houve menor degeneração do epitélio tubular nos animais que receberam glutamina (p=0,029). No fígado, o grupo glutamina apresentou índices menores de tumefação celular do que o grupo controle (p=0,034). No pulmão não houve resultados com significância estatística.

**Conclusão:**

A suplementação prévia de animais experimentais com glutamina via intraperitoneal é capaz de reduzir os danos causados à mucosa intestinal, histoarquitetura dos rins e do fígado.

## INTRODUCTION

Sepsis, defined as a systemic inflammatory response syndrome to infection, is the leading cause of death in Intensive Care Units (ICUs) and one of the leading causes of late hospital mortality, surpassing cancer and myocardial infarction^2^ ^,^ ^3^ ^,^ ^17^.

With the progression of sepsis, three types of changes occur: infection, inflammatory response and hemodynamic change. However, even today the treatment performed is not directly related to the inflammatory response, since it is done only with antibiotics and vasoactive drugs^1^ ^,^ ^3^. In this sense, the action of the amino acid glutamine (Gln) can be very relevant in sepsis, since it would act in the current inflammatory reaction^12^.

Gln is the most abundant free amino acid in plasma and muscle tissue. It is an important source of energy, being used as supplementary energy substrate, since it is an essential component for several metabolic functions, such as: protein and nucleic acid synthesis, nitrogen transport, gluconeogenesis and acid-base homeostasis^20^ ^,^ ^29^. The lower availability of this amino acid may decrease cell resistance to lesions, as there is an imbalance in the Th1 / Th2 response, increased IL-6 secretion in non-hepatic organs and intra-lymphocyte IL-4, and decreased IFN-α expression^29^.

In order for Gln to protect cells from patients submitted to intense metabolic stress, mechanisms such as attenuation in the activation of nuclear factor (NF)-κB, balance between pro- and anti-inflammatory cytokines, reduction in neutrophil accumulation and improvement in intestinal integrity and cellular immune function are used^20^. Preservation of the intestinal barrier occurs through the reduction of intestinal cell apoptosis, making it difficult to pass bacteria through the mucosa^6^. Gln also increases the height of the intestinal villi and the mucosal nitrogen content; stimulates submucosal growth and reduces the accumulation of neutrophils in this layer, preventing multiple organ failure and septicemia^6^ ^,^ ^20^.

Although most experimental and clinical studies highlight the role of Gln in maintaining the intestinal barrier, supplementation with this amino acid is equally effective in other organs. In the liver, there is an increase in hepatic synthesis of antioxidant enzymes, interference in the synthesis of glycogen and fatty acids, in insulin signaling, in protection against apoptosis, especially in the excretion of canalicular biliary acids, as well as trophic potential in hepatocytes^24^. In the lung, there are indications that the use of Gln can prevent the occurrence of injuries, since, under conditions of optimal supplementation, the skeletal muscle and lungs work together to keep the Gln pool in circulation^20^. There is evidence that supplementation of this amino acid causes attenuation of tubular dysfunction and the expression of inflammatory cytokines with the potential for cell injury^21^.

For these reasons, Gln supplementation, in both free and dipeptide form, has been investigated because of its beneficial effects on the clinical evolution of critically ill patients^5^ ^,^ ^20^. In this way, the present study aims to evaluate the effect of Gln in intestine, liver, kidneys and lungs during the period of sepsis.

## METHODS

This study was approved by the Committee on Ethics in Animal Use (CEUA) of the Pontifical Catholic University of Paraná (PUCPR), under protocol no. 893B. The experiments were performed at the Laboratory of Operative Technique and Experimental Surgery of PUCPR. Histological analysis was performed at the Experimental Pathology Laboratory of PUCPR.

### Sample

Twenty male Wistar rats (*Rattus norvegicus,* var. Albinus) were obtained from the animal facility at the Pontifical Catholic University of Paraná, with a mean age of 90 days. The animals were kept in a light/dark cycle (12/12 h), with controlled temperature at 22±1º C and free access to water and food.

The animals were randomly assigned to two groups: Control (n=9) and Gln (n=11).

The animals of the control group were submitted to sepsis by ligation and cecal puncture (LPC), without any previous procedures. On the other hand, animals belonging to the Gln group received dipeptide Gln (L-alanyl L-glutamine-Dipeptiven^®^), with a daily dose of 2 ml/kg (equivalent to 0.4 g of L-alanyl-L- glutamine/kg of rat), intraperitoneally, 48 h prior to induction of sepsis by LPC.

### Anesthesia and operative procedure

The animals were submitted to intraperitoneal anesthesia with ketamine hydrochloride solution (80 ml/kg) and Xylazine 2% (10 ml/kg). Afterwards, the tricotomy of the operative region and fixation of the animal were performed in the supine position at the operative table.

The operation consisted of a median laparotomy with a 3 cm extension. After identification and exposure of the cecum, the fecal milking was performed, ligating with cotton wool 3-0 from 1 cm of the ileo-cecal valve, forming occlusion in a closed pouch of the cecum without, however, causing occlusion of the intestinal transit. Next, a transfixing puncture was performed with a 40x12 mm needle, and soon after, the cecum was placed in the abdominal cavity and the abdominal incision sutured with 3-0 nylon thread. 

### Evaluation of the outcome

Animals were euthanized with administration of thiopental sodium 1 g intraperitoneally, at a dose of 180 mg/kg. After confirming the diagnosis of sepsis, the abdomen was opened for observation of acute peritonitis. The organs were also collected for histological analysis.

### Microscopic evaluation

The removed organs were preserved in formalin 10% for 48 h and posteriorly placed in paraffin, submitted to cross-cut of 4µ with a microtome and stained with hematoxylin-eosin (H&E). The findings were classified by the intensity that was found and transformed in quantitative variables.

The H&E intensity of the findings were analyzed according to the classification: absence or mild presence (0 or 1) and moderate or severe presence (2 or 3).

According to each organ, the findings checked and analyzed were:

Liver - hepatic steatosis, swelling, hepatic sinusoids congestion and presence of intrasinusoidal neutrophils.
Lung - alveolar septum capilar congestion, septum interlobar and interlobular arterioles and arteries congestion, presence of neutrophils in alveolar septum and periarterial neutrophils.
Kidney - glomerular neutrophil, glomerular capilar congestion, tubular epithelium degeneration and intersticial edema.
Gut - intestinal villi lining epithelium desquamation, epithelium neutrophil, edema of lamina propria and inflammation of lamina propria.

### Statistical analysis

The data were analyzed with computer program IBM SPSS statistic for Windows, v 20.0 (Armonk, NY: IBM Corp.). The comparative analysis of control and Gln groups in relation of the variable described was performed using Fisher’s exact test. The significance level used to reject the null hypothesis was p<0,05.

## RESULTS

In the Control group three animals didn’t present sepsis, being excluded from the study (n=6). The Gln group didn’t present loss (n=11). Both groups were submitted to histological analysis.

There were flaws in the analysis of two slides, being that the Control group of Liver and the Gln group of Gut stood with n=5 and n=10, respectively. The remaining stood with Control n=6 and Gln n=11.

**FIGURE 1:**
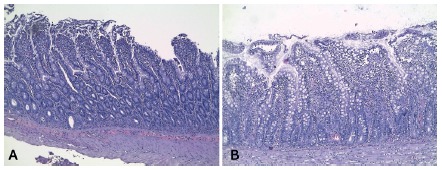
Histological slides stained with H&E showing the epithelial desquamation of the intestine in each group of rats evaluated: A) control group; B) glutamine group.

In the Gut it was verified that the intestinal villi lining epithelium desquamation of the Control group was significantly more intense in comparison to the experimental group (Figure 1) that got the supplement with Gln previously (C 66,7% vs. G 0,0%, p=0,008). The remaining findings didn’t present statistic difference between the groups.

**FIGURE 2:**
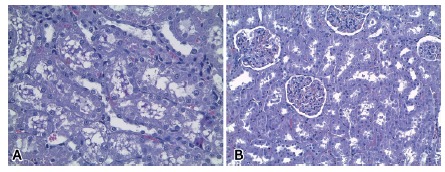
Histological slides stained with H&E showing degeneration of the kidney tubular epithelium in each group of rats evaluated: A) control group; B) glutamine group

As for the renal histological evaluation, there were less moderated to severe degeneration of the tubular epithelium in the Gln group in relation to the Control group (C 50.0% vs. G 0,0%, p=0,029; Figure 2). The remaining parameters analyzed didn’t show statistical difference.

About the hepatic findings, the Gln group presented significant lower rates of intracellular swelling compared to the Control group (C 100,0% vs. G 36,4%, p=0,034). There was no statistical significance on the other findings.

 On the histological tissues of the Lung there were no statistical significance on none of the parameters.

## DISCUSSION

Of all the amino acids present in human organism, Gln is the most affected by conditions which lead to high catabolism. For that reason, in extensive surgeries, wide burn injury and sepsis, Gln tends to be considered a conditionally essential amino acid^12^ ^,^ ^31^.

Low plasmatic concentrations of Gln are related to higher hospital mortality and in intensive care units, once in the course of its depletion both immune system and the gut get severely weakened^22^ ^,^ ^28^ ^,^ ^31^. The benefit of maintenance of plasmatic adequate levels of Gln is also due to its important role on the homeostatic balance with regard to the acid-base balance, nitrogen balance and glucose metabolism^21^.

Besides, it was shown that, rats subjected to endotoxemia through lipopolysaccharide (LPS) administration, the previous treatment with Gln significantly increases the vascular response to vasoconstricting catecholamines, has the potential to decrease of inflammatory cytokines, besides inducting the expression of heat shock proteins, in particular HSP70, that has a vital role in cellular protection and can be detected in many tissues under stress^11^.

The main energetic source to the enterocyte differentiation and proliferation is Gln, besides being a protection factor against apoptosis^19^ ^,^ ^31^. The Gln also exerts an important role preventing bacterial translocation, that is, the passage of microorganism from the intestinal lumen to the systemic circulation, which is related to a decrease of intestinal immunity and to the possibility of evolution to sepsis and multiple organ dysfunction^10^ ^,^ ^23^. One of the explanations to this effect is the Gln’s capacity of stimulating small intestine and colon cell’s trophism^30^.

Ding and Li^7^ conducted a study using rats with trauma or endotoxemia inducted by liposaccharide, which received total parenteral nutrition. It was found that prophylactic treatment with Gln minimizes the increase of permeability of intestinal barrier and bacterial translocation caused by trauma or endotoxemia. Groups that did not receive the additional nutritional contribution did not have great attenuation on the damage caused to the small intestine mucosa. Fabiani e Rocha^8^, by supplementing rats with Gln via enteral for 48 h before the induction of sepsis, observed that there was lower ischemic damage in the small intestine of this animals comparing them to the non-supplemented group. In this study we identified that the intestinal epithelium of the Gln supplemented rats presented lower desquamation and the lamina propria got lower inflammation, in comparison to the control group^7^ ^,^ ^8^.

In sepsis, the kidneys are frequently injured organs, rising critically ill patients mortality. Even if the studies evolving this subject are limited due to difficulty in obtaining histological material and biochemical data of different disease phases, the main pathophysiological theory to explain the renal injury include: injury by ischemia/reperfusion, direct inflammatory injury by cytokine cascades, endothelial cells dysfunction by oxidative stress, coagulation disorder and apoptosis. The progression of this injuries depends on the time that the organ was exposed to sepsis, as low renal perfusion in response of peripheral vasodilatation results in a decreased oxygen demand, leading to tubular epithelium cell injury, apoptosis and acute tubular necrosis^13^ ^,^ ^16^ ^,^ ^25^ ^,^ ^27^.

Oliveira et al.^21^ conducted a study in which they used intravenous Gln in rats submitted to sepsis by the method of cecum ligation and puncture. It was shown that the renal tubular histoarchitecture was restored, showing decrease of renal cell apoptosis, unlike the group that was not treated previously with Gln. In this study, the histopathological evaluation showed lower degeneration of tubular epithelium in rats that got intraperitoneal Gln compared to the control group. As in our study it was evaluated only the histological cuts, parameters as creatinine, urea and other products of the metabolism were not taken into account^21^.

It is believed that there is a relation between hepatic regeneration and Gln supplementation, once the dipeptides overall participate in ureagenesis, gluconeogenesis and protein synthesis^15^ ^,^ ^18^. Magalhães et. al.^15^ verified that Gln suplementation in rats increases the hepatocytes replication in 24 h, being therefore beneficial to hepatic regeneration. The histological analysis in 72 h presented greater number of mitosis in Gln group, as well as changes in histological aspects of hepatic tissue, with hepatocytes proliferation in non-vascularized agglomerates^15^.

This is coherent with the data shown in our study, since there was a decrease in intracellular edema (swelling) in the experimental group in relation to the control group. It is believed that swelling and, therefore, hepatocellular damage, are induced by oxidative stress. Hence the importance of Gln in maintaining cellular reduction to decrease edema^26^. 

We also realized that, though hepatic steatosis and congestion were lower in the group which received Gln, these parameters were also decreased in the control group. That can be explained by the influence of other factors in the regeneration process, as interleukins, intestine derivatives hormones and peptides^9^.

In sepsis, the lungs suffer a great damage. There are fibrin thrombus formation in small pulmonary vessels and an intense inflammatory process in the parenchyma with the formation of vascular congestion, focal bleeding and microabcesses. Consequently, starts the activation of multiple inflammatory and coagulation system pathways contributing to the dissemination of microcirculatory disorders^9^. During this time, one of the most important substances to the protection of the organism is HSP70. In the lungs it acts minimizing the inflammatory process in diseases as acute pulmonary injury^4^ ^,^ ^14^.

The prophylactic Gln administration has the protector effect on the pulmonary parenchyma, because it raises the expression of HSP70 and the number of alveolar macrophages by activating the anti-inflammatory pathway of dephosphorylation CD164/HO-1/p38-MAPK, besides the bronchoalveolar protein concentration and LDH. Li et al.^14^ observed that this treatment attenuated the extension of the edema and pulmonary cell infiltration, and the Western Blot analysis showed a significant increase of HSP in the organ^14^.

In our study, the pulmonary parameters presented did not show statistic relevance that can be justified by the chosen way that the drug was administrated, since for this organ analysis it is recommended to use in inhalation or the intravenous via. Even so, all the groups that received the treatment with Gln had lower intensity in neutrophil infiltration in alveolar septum and periaterial and in arterial congestion and alveolar septum^4^ ^,^ ^14^.

## CONCLUSION

Gln administrated prior sepsis induction in experimental animals showed mitigation to the damage caused to the intestinal mucosa, represented by the fewer epithelium desquamation and fewer lamina propria’s inflammation. In the kidneys; the action of Gln was also evidenced preserving the histoarchitecture of the renal tubules by the smaller degeneration of the tubular epithelium. In the liver, the decrease of intracellular edema suggests that this amino acid has action in intracellular reduction, mitigating in oxidative stress.
